# Educational Escape Room Reinforcement of Infection Prevention in Third-Year Student Pharmacists

**DOI:** 10.3390/pharmacy13050114

**Published:** 2025-08-26

**Authors:** Benjamin Gal, Tony Le, Jiya Thomas, Crystal K. Hodge

**Affiliations:** 1 Department of Pharmacotherapy, University of North Texas Health Science Center System, College of Pharmacy, Fort Worth, TX 76107, USA; 2 Department of Pharmacy, University of Texas Southwestern Medical Center, Dallas, TX 75235, USA

**Keywords:** pharmacy curriculum, gamification, patient safety, infection prevention and control, active learning, pharmacy students

## Abstract

Background: Infection prevention and control (IPC) competencies are essential to safe patient care across practice settings. This cross-sectional survey study aimed to describe the ability of an escape room to reinforce IPC concepts and knowledge retention rates for third-year student pharmacists. Methods: An IPC-themed escape room using a mixture of online and physical puzzles was incorporated into a third-year student pharmacist course. Students in the course took knowledge assessment and perception surveys before the escape room (T1), after the escape room (T2), and for retention at the end of the semester (T3). Results: Statistically significant (*p* < 0.05) increases in knowledge occurred on four out of five of the knowledge assessment questions between the pre- and post-assessments (T1, T2) as well as between the pre- and retention assessments (T1, T3). Student confidence in their ability to provide patient care compliant with IPC practices also demonstrated statistically significant improvement between pre, post, and retention assessments (T1, T2, T3). Conclusions: An IPC escape room is an effective tool to reinforce IPC concepts and increases student pharmacist knowledge and confidence in patient safety practices. Future study iterations should evaluate the role of an IPC IPE event for utility across multiple health professions curricula.

## 1. Introduction

The increasing incidence of hospital-acquired infections (HAIs) and the spread of antimicrobial-resistant microorganisms are considered serious public safety threats by the Centers for Disease Control and Prevention (CDC) [1]. In order to protect the public from the post-antibiotic era and HAIs, the CDC and United States accrediting bodies such as The Joint Commission (TJC) require the implementation of antimicrobial stewardship and infection prevention and control (IPC) strategies in the healthcare setting [1,2]. Within TJC standards IC.01.01.01–IC.03.01.01, a hospital is required to identify risks for acquiring and transmitting infections, design a plan to reduce or eliminate HAIs specifically related to medical equipment, devices, and supplies, prevent practitioner and staff transmission of infectious diseases (ID), evaluate the effectiveness of the plan, and more [3]. As members of the healthcare team and stewardship champions, pharmacists are well poised to implement and collaborate with IPC practices [4]. To corroborate this, the TJC antimicrobial stewardship standard, MM.09.01.01, names both pharmacists and infection preventionists as key members of the antimicrobial stewardship multidisciplinary team [2]. In parallel, the TJC IPC standards also highlight the importance of this relationship by emphasizing antimicrobial stewardship to prevent HAIs [5].

As versatile healthcare team members, student pharmacists must learn, retain, and employ various IPC strategies to practice patient care safely. In order to become a practicing pharmacist in the United States, student pharmacists must meet their state’s criteria for licensure. In general, after student pharmacists graduate with their Doctor of Pharmacy from an accredited program in the US, they must apply for licensure from their state board of pharmacy, pass the general practice knowledge exam titled the North American Pharmacist Licensure Examination, and pass their state’s respective law exam [6]. US pharmacy schools are accredited through the Accreditation Council for Pharmacy Education [7]. Despite the importance of IPC in the aforementioned practice based standards, it is not explicitly listed by the Accreditation Council for Pharmacy Education in the “Required Elements of the Didactic Doctor of Pharmacy Curriculum,” nor within the North American Pharmacist Licensure Examination competency areas [7,8]. The inherent nature of IPC in activities such as sterile techniques for intravenous compounding and universal precautions for immunization training means it is likely that most pharmacy schools teach components of IPC concepts dispersed throughout their respective curricula. To that end, a 2022 survey study of 290 undergraduate pharmacy students in Zambia demonstrated that most student pharmacists had reasonable knowledge, attitudes, and self-reported practices toward IPC measures. However, it is important to note that this was a slightly different student population, as the students were enrolled in a Bachelor of Pharmacy program. Also, the authors noted that Zambia has a high burden of infectious diseases such as tuberculosis [9]. This could have enhanced the students’ appreciation for the importance of IPC practices compared to students enrolled in a US curriculum. Even under this context, the authors argued that pharmacy curricula need more robust IPC practices and programs [9].

In addition to the question of the robustness and ideal placement of IPC skill assessment within pharmacy and various health professions curricula, there is also the question of the best pedagogic approach. Gamification is the use of educational games as an engaging instructional method and has been increasingly studied as an active learning tool in the adult educator’s repertoire [10]. Similarly, a single-blind randomized controlled trial in Saudi Arabia found that an educational board game was statistically more effective than a standard lecture at increasing knowledge scores related to antimicrobial resistance of non-health professionals both after exposure and one month later [11]. A 2015 review article of gamification in pharmacy education provides several examples of real-world use of pharmacy gamification such as “Who Wants to Be a Med Chem Millionaire”, PK poker, board game adaptations, and more [10]. Another example of gamification is the escape room, where participants are tasked with solving educational puzzles to “escape” from a room. Prior medical, nursing, and pharmacy education literature have shown the benefit of using an escape room as a teaching method [11,12,13,14,15,16,17,18].

Student pharmacists’ escape room educational topics have varied from interprofessional teamwork, diabetes care, non-sterile compounding, good manufacturing practices, and more [17,18,19,20,21]. There is also a multi-center, quasi-experimental study comparing pre and post-scores of emergency medicine residents who either underwent a virtual escape room or traditional lecture (s) related to ID. Residents who participated in the virtual escape room showed a statistically significant improvement in knowledge scores, while those who attended a lecture did not [16]. This study contributes to the premise that escape rooms can teach ID-related content, such as IPC. This assertion is also supported by nursing student literature using gamification to reinforce patient safety and quality improvement, which incorporates IPC components [15,22].

The authors are unaware of literature specifically assessing US student pharmacist ID or IPC content as an escape room or retention of applicable knowledge gains from escape rooms. An escape room prior to advanced pharmacy practice experiences (APPEs), where patient care exposure is highest, poses an opportunity within a pharmacy curriculum to use gamification as reinforcement of IPC techniques and concepts. The purpose of this single-center, cross-sectional pilot study was to assess the ability of an educational escape room to improve IPC knowledge of third-year student pharmacists (P3s) across three time periods: before the escape room, after the escape room, and at the end of the semester.

## 2. Materials and Methods

### 2.1. Study Design and Setting

This study was approved as a sub-study of a larger educational protocol approved by the North Texas Regional Institutional Review Board for studying educational activities within a single institution’s Doctor of Pharmacy curriculum. The design of this study was a single-center, cross-sectional survey study of student pharmacists who participated in the escape room activity incorporated into a fall 2019 P3 recitation course. At the time, educational gamification, including the use of escape rooms, was starting to be increasingly studied in US health professions programs and primarily focused on pre- and post-assessments for efficacy [13,18,20]. The course faculty wanted to determine if an IPC escape room could be used as an interprofessional education (IPE) activity, mirroring the importance of the topic as noted in the aforementioned TJC standards [2,3,4,5]. The educational activity was an instructor developed IPC escape room initially designed to be a student pharmacist pilot. If the IPC escape room was assessed to be successful, it would be considered for direct incorporation into the health science center IPE curriculum. However, due to pharmacy curricular changes and the pandemic, the educational activity and corresponding survey study were only implemented in 2019.

Appropriate timing for implementation within the pharmacy curriculum was identified to be the P3 year. The rationale was that by this point in the curriculum, student pharmacists have adequate exposure to IPC practices by completing immunization training, sterile compounding, community introductory pharmacy practice experiences (IPPEs), and hospital IPPEs. The fall semester, approximately August–December in this bi-semester program, of the P3 year was also when students were concurrently enrolled in a four-credit hour, semester-long ID integrated pharmacotherapeutics (IPT) course and a one-credit hour recitation course designed to apply didactic content learned from IPT courses.

The escape room was directly incorporated into the recitation course as a required session; all P3 student pharmacists enrolled in the recitation course participated in the activity. Team-based learning (TBL) activities are a regular component of this school’s curriculum, including the recitation course where the escape room occurred. By utilizing assigned groups for TBL activities, the time allotted for relational trust essential to team success was minimized. For this cohort, there were 14 groups for TBL, ranging in size from five to seven students. The escape room activity was conducted in their designated groups, while all surveys were completed on an individual basis. Students had approximately two hours to complete all escape room activities during the session. Successful completion of the escape room did not impact student grades and no group ended up needing extra time. The study research objectives included: (1) assess the ability of an escape room to improve IPC knowledge, (2) evaluate retention of IPC knowledge, and (3) describe the impact of an escape room on student confidence to apply IPC practices to patient care.

### 2.2. Escape Room

#### 2.2.1. Escape Room Design and Assessments

The escape room was designed as a student pharmacist pilot for later implementation as a broader IPE event via synchronous, in-person activities where students apply IPC standards. Student objectives for the escape room included (1) implementation of basic IPC practices in an inpatient case simulation, (2) facilitation of transitions of care through an emphasis on interprofessional collaboration, and (3) an analysis of critical errors that contributed to patient HAIs. Prereading materials were identified by course faculty and posted on the course Canvas™ page with guidance to review before completing the pre-knowledge and perception surveys. Canvas™ is the institution’s learning management system.

Pre and post-surveys to assess knowledge and perceptions have previously been described to assess the effectiveness of escape rooms as an educational tool in US pharmacy curricula [13,18]. In these examples, pre-assessments were conducted 1–3 weeks prior to the escape room, then a post-assessment was administered either as the last activity of the escape room or immediately afterward. The assessments for this escape room were similarly designed by course faculty as pre- (T1), post- (T2), and retention (T3) assessments to measure student knowledge acquisition and perceptions specific to IPC. A unique, one-time student Qualtrics™ link was emailed to students for both knowledge and perception assessments (questions available in Appendix A). The knowledge assessment was a five question, multiple choice quiz graded for accuracy. The questions were the same across all time points. The perception survey was graded for completion and consisted of eight Likert scale questions from strongly agree, agree, disagree, and strongly disagree. Completion grades for perception surveys were utilized to incentivize responses while minimizing response bias. Students were informed that the course director and session instructor were blinded to individual perception survey responses but had access to coded and aggregate responses for analytical purposes. The pre-knowledge and perception surveys were due prior to the activity (T1), which was held in August of 2019. The post-knowledge and perception surveys were also administered through unique, one-time Qualtrics™ links and were due within a week after the activity (T2). At the end of the semester, approximately three months after the escape room activity, the knowledge and perception surveys were again administered to assess retention (T3). Therefore, students took a total of six assessments related to the required IPC escape room.

#### 2.2.2. Patient Cases

Students were instructed that the goal of the escape room was to help the simulated patient cases be appropriately discharged from the hospital without a HAI. Success in the escape room did not impact the student’s grade for the session. The same two patient case scenarios were provided to each TBL group with predetermined updates to one or both patient cases after completion of escape room puzzles. Patient case, MJ, was a 73-year-old female admitted from a skilled nursing facility for a stroke. She had a notable past medical history of Barrett’s esophagus and prior infection with an extended-spectrum beta-lactamase *Klebsiella pneumoniae* treated with ertapenem within the last two months. Patient case, PP, was a 26-year-old male admitted to the intensive care unit for a gastrointestinal bleed. His past medical history included chronic migraines and an old sports injury that limited his range of motion. Activities were a mix of online puzzles and physical activities. Table 1 contains more detailed puzzle information.

After each puzzle, the student group would receive information about the next step in MJ and PP’s care, including room transfers, procedures such as endoscopies, various healthcare team member interactions such as physical therapy, and more. Progression of the case would then prompt the students to the next puzzle.

During the escape room, previously trained faculty, students on APPEs with faculty, and student navigators were available to facilitate any difficulties with puzzle instructions or completion as needed. At the start of the session, students were warned that professionalism points could be deducted at the discretion of the facilitators. No professionalism points were ultimately deducted during the implementation of the escape room. Since the cases were designed for the simulated cases to develop an HAI during the escape room, the last activity was a large group, class-based analysis of how the simulated patients acquired their HAIs and what preventative steps could have been taken to avoid their respective HAIs.

#### 2.2.3. Faculty and Facilitator Activities

This escape room was designed as a pilot activity within the pharmacy curriculum with the potential for later implementation as a larger IPE event. Therefore, an interprofessional team of ID pharmacy faculty, recitation course coordinators, representatives from the physician and physician assistant programs, and a member of the IPE office collaborated to create a storyboard of the escape room and brainstorm activities. The group agreed upon practical techniques and key points, emphasizing TJC IPC standards to include in patient cases or activities as appropriate. Online activities were integrated into the recitation course Canvas™ page. Knowledge assessments and perception surveys were developed and administered through Qualtrics™. Results from the Qualtrics™ assessments and surveys were de-identified by using a unique student code to maintain blinding. The student navigator was the only one with the code key and translated grades to Canvas™. The principal investigator, CKH, supplemented physical puzzles and supplies when materials from other lab-based courses were not available (e.g., chocolate syrup and tarp).

A one-hour facilitator training session occurred approximately the week before the escape room activity. During the training, case progression, puzzle and activity answers, and objectives were reviewed. Anticipated points of difficulty were identified with guidance for facilitation, including appropriate resources to provide to students. Since some of the facilitators did not have a clinical background, the escape room answer key provided multiple potential interactions with students and navigation tips. Facilitators were also provided with the answer key to the knowledge assessment. Additionally, two volunteer facilitators were trained to apply chocolate syrup to student gowns and gloves as part of the student doffing puzzle described in Table 1. During the activity, there were approximately six facilitators for the 14 groups of TBLs. The escape room event was conducted in a single large classroom with large group tables along the sides of the room, allowing the facilitators to easily monitor and support their designated groups. All activities except for the doffing puzzle with chocolate syrup could be conducted at the TBL group’s table; the doffing puzzle had a designated area at the end of the room over a tarp.

### 2.3. Statistical Analysis

For research purposes, only data from students who completed all pre-activity (T1), post-activity (T2), and end of semester (T3) surveys were analyzed in order to perform paired statistics. A sample size to prevent type II error was not calculated since the activity was meant to serve as a pilot and was implemented, similar to other literature, as a required course activity [13,18]. The knowledge assessment was five questions and required an 80 percent or more to be considered passing. The mean percent correct for each question on the knowledge assessment was calculated for the time periods before the escape room (T1 or pre), after the escape room (T2 or post), and at the end of the semester (T3 or retention). Knowledge assessment results were analyzed using the Cochran Q test to determine if a significant difference existed in the number of questions answered correctly between the three time points. The McNemar test was used for pairwise comparisons if the Cochran Q test showed significance. Descriptive statistics, including mean, median, and mode, were conducted for the percentage of students who passed the knowledge assessment at each time point. Perception survey results were ordinal comparisons of strongly disagree, disagree, agree, and strongly agree. Given the anticipated small sample size of a pilot study, the category of “not applicable” or “no difference” was not provided as an option. At each time point, the percentage of students that selected each agreement category was recorded. The Friedman test was then used to determine significant differences in rankings of survey results between the three time points. The Wilcoxon signed rank test was utilized for pairwise comparisons if the Friedman test showed significance. No corrections for multiple analyses or sensitivity analyses were conducted. Significance was defined a priori as a *p* < 0.05. Statistical analysis calculations were set up and performed using Microsoft Excel^®^ and confirmed with IBM SPSS^®^ Statistics version 27.0, 6 July 2020.

## 3. Results

All 85 students in the course took part in the escape room activity, 66 completed the knowledge assessment at all three time points and 68 students completed the perception survey at all three time points. Significantly more students passed the post-escape room knowledge assessment (T2 mean, 100%) than the pre-escape room assessment (T1 mean 39%; *p* < 0.001); significantly more students passed the retention knowledge assessment (T3 mean 92%) than the pre-assessment (T1 mean 39%: *p* < 0.001). Significantly more students answered correctly on the post (T2) and retention (T3) knowledge assessments than on the pre-escape room (T1) questions pertaining to IPC precautions, airborne Personal Protective Equipment (PPE), handwashing technique, and doffing PPE (questions 1,3, 4, and 5 on Table 2).

Question two, regarding the appropriate use of alcohol-based sanitizer, had no statistically significant difference in correct student responses between the three time points. For questions with a statistically significant Cochran Q test, pairwise comparisons were performed and are listed in Table 3. There was a statistically significant decrease in the percentage of students who correctly answered question four related to hand hygiene techniques between the post and retention assessments (T2, T3).

On the perception survey, student confidence had a statistically significant increase in the percentage of student agreement categories between the pre, post, and retention surveys (*p* < 0.001). Student responses to questions on their perception of interprofessional collaboration, working in groups, and attitude towards educational escape rooms did not significantly change between the three time points. On all three surveys, most students indicated positive attitudes towards interprofessional collaboration (questions three and five) and the use of escape rooms (questions one, six, and eight). Perception survey results at all three time points are shown in Table 4.

## 4. Discussion

The results from this study demonstrate that an escape room activity improves students’ knowledge and confidence in IPC concepts. To the authors’ knowledge, this is the first escape room study of US student pharmacists with retention assessed more than one month after the escape room. While there was a decrease in the percentage of students who answered correctly between the post-escape room (T2) and retention (T3) assessments on questions 1, 4, and 5, a net benefit still occurred. This is evinced by the significant increases in percent correct between pre-escape room (T1) and retention (T3) assessments on four out of the five knowledge questions. The significant increase in students passing the knowledge assessment further highlights the benefit of an escape room activity to reinforce IPC concepts. Overall, the results of the knowledge assessments align with previous literature, indicating that an escape room is an effective educational technique [17,18,23]. The results of this study, nursing literature, and the previously mentioned escape room for medical residents support the hypothesis that an escape room can effectively reinforce IPC concepts [15,16,22]. IPE escape rooms related to sepsis and acute care have demonstrated increased content knowledge and teamwork amongst participating medical, nursing, pharmacy, and physical therapy students [14]. Given the ubiquitous importance of IPC throughout healthcare, it may be beneficial to implement an IPC educational escape room as an IPE activity involving several health professional schools prior to their respective clinical experiences.

Contrary to this study, Clauson et al. did not demonstrate a statistically different improvement of overall APPE readiness of P3s after an escape room [13]. There are at least two significant contributors to this discrepancy. The first of which is the extent of student pre-escape room knowledge. Clauson et al. sought to determine if their P3 student pharmacists were APPE-ready and had a high baseline mean pre-escape room score of 81%, indicating that a significant number of the student pharmacists were prepared for APPEs prior to the activity [13]. Despite not being explicitly listed by accrediting bodies for incorporation into pharmacy curricula, IPC concepts such as proper PPE, handwashing, and correctly identifying types of precautions should be second nature to student pharmacists, particularly by the P3 year [7]. At that point, student pharmacists should have been exposed to IPC concepts throughout the curriculum and experienced aspects of IPC while on IPPE rotations. Surprisingly, this did not lend itself to a high level of baseline knowledge, such as that seen by Clauson et al., with only 39% of student pharmacists in our study passing the pre-escape room (T1) knowledge assessment. A second contributor that likely explains the difference between the studies is the content itself. The escape room in Clauson et al.’s study was broader in scope, as it was an end of P3 year assessment of readiness for APPEs [13]. This study was limited to IPC and IPE in scope, which highlights a potential gap and resulting need for summative reinforcement of IPC in pharmacy curricula, at some point in the P3 year prior to APPEs.

Encouragingly, student confidence gains paralleled the increased success rates seen on the knowledge assessment, suggesting an appropriate correlation between actual knowledge growth and confidence in implementing IPC strategies. Most student pharmacists’ attitudes towards the escape room activity, interprofessional collaboration, and group work were generally positive, independent of the IPC escape room activity, and may explain why there was no difference detected. This study’s College of Pharmacy is part of a health science center that houses several healthcare professional programs that frequently collaborate in IPE activities. The high exposure to IPE activities and frequent promotion of teamwork may account for baseline positive perceptions of collaborating in interprofessional teams. Furthermore, students in this cohort completed an educational escape room on diabetes as part of their second-year curriculum. Prior exposure to educational escape rooms may have influenced their initial perceptions of the effectiveness of educational escape rooms. Our results correlate with previous studies that indicate that students enjoy educational escape rooms and find them beneficial tools for learning and enhancing teamwork [18,23].

While this study was novel in assessing retention and IPC incorporation, an additional strength of this study is the inclusion of significant methodologic detail on human resources and facilities. As a pilot program for eventual transition to an IPE, faculty and resource accountability are important considerations for sustainability. The use of clinical and nonclinical facilitators expanded the availability of human resources to support the activity with an approximate ratio of 1 facilitator to 2 student groups. Instructor time was also needed to develop a facilitator guide and the training materials for activity facilitators. This additional information will aid replicability and sustainability when implementing future iterations and in multiple health professions.

This study also has its limitations. The first of which is that, in addition to no existing standard for escape room implementation, the authors are not aware of a validated survey available to evaluate the efficacy and perceptions of escape rooms in health professions curricula. While inspiration was drawn from previously published reports of escape rooms, the course instructors de novo created all surveys and assessments used in this study, which impacts the generalizability of the study [13,18]. Additionally, the cross-sectional study design does not prove causality and only demonstrates an association between the activity and improved scores for that particular session. No comparisons to the ID IPT course or recitation course grades were made and could have potentially confounded the results. Additional potential confounders that may have affected study results include varying exposures to IPC practices during IPPEs and prior exposure to escape rooms. In addition, the ID IPT was ongoing throughout that semester and may have confounded the results, especially the retention (T3) knowledge assessments. While the ID IPT did not have a dedicated session to IPC, some relevant session content (ex: *Clostridioides difficile* infection and contact precautions with handwashing) was incorporated. This may have contributed to the retention of IPC concepts at the end of the semester. However, including an escape room in the same course or temporally adjacent material is consistent with escape room assessments incorporated into a specific course including, but not limited to, escape rooms on diabetes, geriatric pharmacy, and non-sterile compounding [12,18,23,24].

Additionally, while this pilot can be used to support the idea that knowledge assessments administered months after an educational intervention, such as gamification, can be used to assess knowledge gain retention, this study was not designed to prove retention causality. As a novel component of the assessment of escape rooms, future large-scale studies are needed to test the validity of retention post-event knowledge assessments as a pedagogical tool and compare the validity and efficacy to traditional methods. Additionally, future qualitative assessments such as focus groups may be helpful in delineating unknown potential confounders, barriers, and facilitators in retention assessments and the activity’s potential as an IPE activity.

An additional limitation of this study is the small sample size. The study was limited to one class of student pharmacists at a single institution due to the desire to evaluate the benefits of a pilot program before expanding to a larger IPE activity, curricular changes within the pharmacy program, and pandemic restrictions. A priori discussions with the IPE office determined that if this study in student pharmacists demonstrated efficacy in increasing knowledge and confidence, the IPC escape room might be adapted as a required IPE activity within the curricula of multiple schools on the health science center campus. Another reason for the sample size is that the study only included results from students who completed the assessment at all three time points (pre, post, retention). Of the three time points, the least number of students completed the end of semester surveys assessing retention (T3), substantially contributing to the limited sample size. This was likely because the final assessment was administered when students were studying for finals in a one credit hour course and may represent a lower student priority [13]. The timing also correlated with respiratory virus season, during which several health organizations, like the CDC and World Health Organization, routinely heavily promote handwashing. Discrepancies between recommendations for the proper duration of handwashing by these organizations exist, which may account for the statistically significant decrease in students who answered this question correctly between the post (T2) and retention (T3) knowledge assessments [25,26,27]. Despite the small size, there were still statistically significant improvements in four out of the five questions on the knowledge assessment, negating the need for a larger sample size to answer those questions.

Although this study focused on student pharmacists, the findings from the study are likely to be applicable to other health professions as well. A quasi-experimental design study directly comparing the efficacy of an escape room to a traditional lecture and a randomized controlled trial comparing a board game to conventional lecture support this assertion [11,16]. This study and previous studies show that educational escape rooms are an effective and enjoyable way to teach didactic content [12,14,20,23]. Translational science of gamification comparing subsequent rates of practice implementation and patient-centered outcomes are warranted.

## 5. Conclusions

The use of an educational escape room successfully increased student pharmacists’ immediate and three-month knowledge and confidence in implementing IPC practices. This study was a pilot study for an IPC IPE initially implemented within the pharmacy curriculum. The results of which may support the incorporation of activities like educational escape rooms to reinforce IPC knowledge within health professions curricula. Future study iterations should evaluate the role of an IPC IPE event across multiple health professions and further compare retention assessments from gamification to traditional pedagogy methods.

## Figures and Tables

**Table 1:** Escape room puzzle information.

Activity Objective	Puzzle Environment	IPC Concept
Use an antibiogram to answer three questions	Online	Describe local resistance rates
Identify risk factors for resistance for MJ	Online	Identify risk factors for resistance and the need for isolation
Determine the utility of an MRSA nasal screening test and application of the results to a patient’s risk of a MDRO	Online	Determine colonization with MDROs and implications for practice
Crossword puzzle of IPC terms including: disinfection, sterilization, contaminated, cleaning, terminal room cleaning, PPE, HAI, and nosocomial infection	Online	Review common definitions and prevention practices
Donning/doffing practice: two students from the group will don contact precautions PPE in front of a facilitator, have chocolate syrup applied to their gowns, then appropriately doff without transferring the chocolate syrup	Physical	Donning and doffing of PPE and visualization of potential for contamination
Verbalize to a facilitator the appropriate timing for when PPE should be removed	Physical	Doffing and preventing contamination of clean areas
Verbalize and demonstrate hand hygiene for both soap/water and hand sanitizer	Physical	Hand hygiene
QR code matching of universal, contact, droplet, airborne, and enteric precautions with appropriate PPE materials	Physical	Different types of PPE terms and materials

HAI = hospital-acquired infection; IPC = infection prevention and control; MDRO = multidrug-resistant organism; MRSA = Methicillin-resistant *Staphylococcus aureus*; PPE = personal protective equipment; QR = quick response.

**Table 2:** Knowledge assessment pre- (T1), post- (T2), and retention (T3) percent correct.

Knowledge Assessment Question Concept	Pre (T1) Correct (%)	Post (T2) Correct (%)	Retention (T3) Correct (%)	*p* Value
Q1. Pair IPC precautions with appropriate PPE	47	97	89	<0.001
Q2. Identify when alcohol-based sanitizer is insufficient hand hygiene	94	100	100	NS
Q3. Choose the appropriate PPE for airborne isolation	88	98	100	<0.001
Q4. Determine the appropriate order for hand hygiene steps	50	100	77	<0.001
Q5. Recognize appropriate timing for doffing disposable gown PPE	47	98	89	<0.001

NS = Not significant; PPE = personal protective equipment.

**Table 3:** Knowledge assessment *p*-values for differences between time points.

Question	Pre-Post (T1–T2) *p*-Value	Pre-Retention (T1-T3) *p*-Value	Post-Retention (T2–T3) *p*-Value
Question 1	<0.001	<0.001	NS
Question 3	0.02	0.01	NS
Question 4	<0.001	0.005	<0.001
Question 5	<0.001	<0.001	NS

NS = Not significant.

**Table 4:** Perception survey results.

	Student Answer											
	Strongly Disagree (%)			Disagree(%)			Agree (%)			Strongly Agree (%)		
Question Topic	Pre (T1)	Post (T2)	Retention (T3)	Pre (T1)	Post (T2)	Retention (T3)	Pre (T1)	Post (T2)	Retention (T3)	Pre (T1)	Post (T2)	Retention (T3)
Recommend use of escape room for retention	3	6	10	18	10	4	53	40	46	26	44	40
Confidence in ability to provide care compliant with IPC guidelines	3	1	3	26	7	1	57	57	62	13	34	34
Importance of interprofessional collaboration in keeping patients safe from HAIs	1	0	1	0	0	0	22	28	32	76	72	66
Difficulty focusing due to multiple components of escape room	7	15	9	37	35	35	50	29	35	6	21	21
Preference for working in groups	1	4	1	24	13	24	54	57	51	21	25	24
Enjoyment of games to facilitate learning	0	4	3	13	7	12	46	43	41	41	46	44
Previously completed an escape room	1	3	3	4	1	1	41	34	53	53	62	43
Recommend activity to learn HAI prevention or patient safety	0	7	7	15	16	7	65	35	46	21	41	40

HAI = hospital-acquired infection; IPC = infection prevention and control.

## Data Availability

The original contributions presented in this study are included in the article material. Further inquiries can be directed to the corresponding author.

## Abbreviations

The following abbreviations are used in this manuscript:

APPE	Advanced Pharmacy Practice Experiences
CDC	Centers for Disease Control and Prevention
HAI	Hospital-Acquired Infection
ID	Infectious Disease(s)
IPC	Infection Prevention and Control
IPE	Interprofessional Education
IPPE	Introductory Pharmacy Practice Experiences
IPT	Integrated Pharmacotherapeutics
MDRO	Multidrug-Resistant Organism
MRSA	Methicillin-Resistant *Staphylococcus aureus*
NS	Not Significant
PPE	Personal Protective Equipment
P3	Third-year student pharmacists
TBL	Team-Based Learning
TJC	The Joint Commission

## Appendix A

### Appendix A.1. Knowledge-Based Survey Questions

1 Which of the following is correctly paired?

- Hand hygiene: universal precautions
- Hand sanitizer: enteric precautions
- Gown: respiratory precautions
- Respiratory masks: airborne precautions

2 When should you NOT use an alcohol based sanitizer for hand hygiene?
3 After direct patient interaction
4 After removing gloves
5 Prior to direct patient interaction
6 When hands are visibly soiled
7 Which of the following Personal Protective Equipment (PPE) is required for an airborne isolation room?

- Dedicated stethoscope
- Disposable gown
- Gloves
- N95 mask

8 Which of the following represents the appropriate way to wash hands?

- Apply water -> apply soap -> rub hands vigorously covering all surfaces for at least 15 s -> rinse hands with water -> dry hands
- Apply water -> apply soap -> rub hands vigorously covering all surfaces for at least 20 s -> rinse hands with water -> dry hands
- Apply soap -> apply water -> rub hands vigorously covering all surfaces for at least 15 s -> rinse hands with water -> dry hands
- Apply soap -> apply water -> rub hands vigorously covering all surfaces for at least 20 s -> rinse hands with water -> dry hands

9 When should a disposable gown (PPE) be doffed?

- After exiting a patient’s room
- After writing your SOAP note on a patient
- Prior to entering the patient’s room
- Prior to exiting a patient’s room

### Appendix A.2. Perception Survey Questions

Please indicate which of the following most closely relates to your perception of the infection control and prevention escape room.

**Statement**	**Strongly Agree**	**Agree**	**Disagree**	**Strongly Disagree**
I recommend the use of escape rooms as an activity to improve retention of content.				
I feel confident in my ability to provide care in a way that is compliant with infection control and prevention recommendations and/or guidelines.				
I believe interprofessional collaboration is imperative in keeping patients safe from hospital-acquired infections.				
It was/will be difficult for me to focus on learning because of the multiple components of an escape room.				
In general, I like working in groups.				
In general, I enjoy playing games to facilitate learning.				
I have done an escape room before today.				
I recommend this activity for learning to prevent hospital-acquired infections and/or the importance of patient safety.				
